# Arts-Based Research Approaches to Studying Mechanisms of Change in the Creative Arts Therapies

**DOI:** 10.3389/fpsyg.2018.02076

**Published:** 2018-11-01

**Authors:** Nancy Gerber, Karolina Bryl, Noah Potvin, Carol Ann Blank

**Affiliations:** ^1^Department of Creative Arts Therapies, Drexel University, Philadelphia, PA, United States; ^2^Department of Art Education, Florida State University, Tallahassee, FL, United States; ^3^Mary Pappert School of Music and School of Nursing, Duquesne University, Pittsburgh, PA, United States

**Keywords:** mechanisms of change, transformation, psychotherapy, creative arts therapies, arts-based research

## Abstract

The purpose of this preliminary qualitative research study is to explore the role and function of multiple dynamic interactive aesthetic and intersubjective phenomena in the creative arts therapies process relative to transformation in perception, behavior, relationship, and well-being. A group of doctoral students and faculty studied these phenomena in an analogous creative arts therapies laboratory context using a method called *Intrinsic Arts-Based Research. Intrinsic Arts-Based Research* is a systematic study of psychological, emotional, relational, and arts-based phenomena, parallel to those emergent in the creative arts therapies, using individual and collective intrinsic immersive and reflective experience in combination with qualitative and arts-based research methods. Our primary goal was to simulate the creative arts therapies experience in order to identify, document, and describe the complex transformative phenomena that occur at the nexus of arts-based expression, reflection, and relationships in the arts therapies. For the purposes of this paper transformation is defined as “…. a significant reconfiguration of perception and thought resulting in the lessening of psychic restraint and pain, allowing for the emergence of new psychological perspectives that contribute to living a more creative life” (Gerber et al., 2012, p. 45). Through a deductive thematic analysis of written accounts of these simulated creative arts therapies experiences by participant/researchers in the laboratory we identified three primary dynamic and interactive broad constructs that together, with more specific modifying themes, might account for and describe change within the creative arts therapies. These broad dynamic interactive themes are: ruptures, resolutions, and transformation; relationship and intersubjectivity; and, arts-based expressive processes. The more specific modifying themes include: *dialectical rupture and resolution, relational attunements and ruptures, imaginational flow, transcendence and ruptures, sensory/kinesthetic/embodied ways of knowing, and intersubjective transcendence*. We propose that change in the creative arts therapies is driven more by a dynamic system of interactive phenomena the varying combinations of which create conditions for relational attunement, imagination, dialectical tensions and creative resolutions, and the ultimately creative transformation.

## Introduction

“*In the experience of art we see a genuine experience… induced by the work which does not leave him who has it unchanged… so we hope to better understand what kind of truth it is that encounters us there” (Gadamer, 1975/2003, p. 100)*.

The purpose of this preliminary qualitative research study is to explore the role and function of multiple dynamic aesthetic and intersubjective phenomena in the creative arts therapies that might be considered mechanisms of change. A group of doctoral students and faculty have been studying these phenomena in an analogous creative arts therapies laboratory context using a method called *Intrinsic Arts-Based Research. Intrinsic Arts-Based Research* is a systematic study of psychological, emotional, relational and arts-based phenomena using individual and collective intrinsic immersive and reflective experience in combination with qualitative and arts-based research methods. Our primary goal is to simulate the creative arts therapies experience in order to identify, document, and describe the complex transformative phenomena that occur at the nexus of arts-based expression, reflection, and relationships in the arts therapies. For the purposes of this paper transformation is defined as “…. a significant reconfiguration of perception and thought resulting in the lessening of psychic restraint and pain, allowing for the emergence of new psychological perspectives that contribute to living a more creative life” (Gerber et al., 2012, p. 45).

Since the beginning of this project 8 years ago, we have continually engaged in an ongoing critical reflection and evaluation of our underlying philosophical assumptions about the nature of reality and knowledge for our creative arts therapies fields. Through the examination of our philosophical assumptions we created and adopted an aesthetic intersubjective paradigm (Chilton et al., 2015). This worldview is predicated upon the philosophical assumptions that our perceptions, relationships, and behavior are conceived and reside in dynamic co-constructed pluralistic intersubjective realities in which an aesthetic epistemic comprises the knowledge and communication. We define *aesthetics* as pre-verbal sensory-based, embodied perceptual and imaginal knowledge that emerges and acquires meaning in intersecting historical and current intersubjective narratives (Cooper, 1997; Harris-Williams, 2010; Brown, 2011; Chilton et al., 2015). Intersubjectivity is defined as a pre-verbal unconscious phenomenon wherein “jointly constructed narrative… ascribes meaning to experience for which no language previously existed” (Brown, 2011, p. 1) and “communication and meaning making between two intrapsychic worlds… results in changes within each member…” (Brown, 2011, p. 109). Intersubjectivity emphasizes the shared lived experience in which heightened empathy and attunement allows one to enter the emotional experience of another in order to co-construct a new, re-imagined, and often transformative life narrative (Stern, 2005).

These ontological and epistemic foundations of the creative arts therapies represent the archeology of the most profound human emotional and relational constructs essential to understanding the nuances and complexities of the human experience. Implicit in the creative arts therapies worldview is that aesthetic intersubjective ways of being and knowing exist on the periphery of consciousness inaccessible through traditional investigative methods or verbal discourse. In creative arts therapies practice we use our arts forms to elicit the expression of these most profound experiences, construct personal narratives, and enhance self-awareness within a carefully constructed and emotionally held relationship; while in research, we use arts-based methods for purposes of systematic inquiry into creative arts therapies phenomena.

Based upon our adopted worldview and our objectives to study transformative processes in the creative arts therapies, we selected a comparable arts-based research philosophical and methodological approach to investigate these complex aesthetic intersubjective human phenomena. The arts-based research approach we adopted is one in which the arts are used as the primary method of systematic investigation and analysis throughout the research process (Hervey, 2000; McNiff, 2008; Kossak, 2012; Viega, 2016) “…as a primary way of understanding and examining experience…” (McNiff, 2008, p. 29) in the study of the multi-dimensional psychological and socio-cultural human condition (Gerber and Myers-Coffman, 2017). Furthermore, Barone and Eisner (2012) assert that “[arts] based research is an effort to extend beyond the limiting constraints of discursive communication in order to express meanings that otherwise would be ineffable” (1).

To implement our arts-based research study of the therapeutic and transformational phenomena in the creative arts therapies we developed a creative arts therapies laboratory in which a group of doctoral students and faculty simulated the creative arts therapies and studied the parallel individual and collective arts-based intersubjective processes. We created and used what we call an intrinsic arts-based research method (Hagman, 2005; Levine, 2005; Gerber et al., 2012; Chilton et al., 2015; Gerber and Scotti, 2017). Within the intrinsic arts-based research approach, we used ourselves as participant/researchers to study arts-based relational phenomena as they emerge organically within the intersubjective context paralleling the creative arts therapies process. As participant/researchers, we navigated between the immersive arts-based intersubjective process and reflective analytic procedures documenting our experiences through arts-based expressions, reflective journaling, group discussions, qualitative and arts-based data analysis. The results of our inquiries were analyzed, synthesized, and documented in culminating textual and arts-based projects at the conclusion of each academic term with a retrospective summative analysis at the end of the year.

This article represents a preliminary qualitative analysis of a sampling of these retrospective culminating projects written by doctoral student participant/researchers over the past 8 years who sought to answer the question: “What are the factors that contribute to therapeutic mechanisms, psychological understanding, meaning making, and transformation within the intersubjective arts therapies process?” in this creative arts therapies laboratory course (Gerber et al., 2012; Gerber and Scotti, 2017).

In this preliminary phase of the project we have randomly selected eight retrospective de-identified study records representing student culminating projects from the creative arts therapies laboratory course and adopted a deductive or theoretical thematic analytic approach (Braun and Clarke, 2006) to study patterns of evidence relative to transformative experiences. We selected this approach for the explicit purposes of developing and evaluating a coding system based upon exogenous research and theory about transformative phenomena to compare to the heuristic data generated from our intrinsic arts-based and qualitative investigations. Our aim was to determine how our emergent intrinsic phenomena aligned with extrinsic empirical mechanisms of transformation to further understand what creative arts therapies processes contribute to change. To identify deductive thematic concepts for our study, we conducted a review of the literature focused on the definitions of mechanisms of change in general, and mechanisms of change in psychotherapy and the creative arts therapies.

In contemporary scientific research, particularly within the domains of medicine and the physical sciences, mechanisms of change are defined as causal and measurable variables that statistically account for the relationship between a particular therapeutic intervention and outcome (Kazdin, 2007). Kazdin and Nock (2003) stated that mechanisms of change not only represent the causal relationship but also “reflect the processes through which therapeutic change occurs” or “those processes or events that lead to and cause therapeutic change” (1117). According to Kazdin (2007) mechanisms are evaluated based upon principles of association, plausibility, consistency, experimental manipulation, timeline, and gradient (Kazdin, 2007). Petrik and Cronin (2014, p. 284) resonate with this definition but add that in psychotherapy mechanisms are the “theory driven reason that change occurs in therapy or the *how* or *why* of the therapeutic change.” They add, in addressing mechanisms of change in psychotherapy, that the mechanisms inhabit the dynamic interaction between technique, client-therapist processes, and outcomes.

Mechanisms are interconnected with moderators, which are pre-existing and co-existing conditions, and mediators, which are other intervening variables that influence the causal mechanistic effect (Kazdin and Nock, 2003; Johansson and HØglend, 2007; Kazdin, 2007). A moderator is considered to be a “pre-treatment variable” that relates to “for whom and under what conditions the effects will occur” (Johansson and HØglend, 2007, p. 2) such as gender, illness severity, genetic pre-dispositions, family, medical and psychological history, as well as social constructs such community and culture (Kazdin and Nock, 2003, p. 1118; Johansson and HØglend, 2007). A mediator is an “intervening variable” (Kazdin, 2007, p. 3) that represents processes occurring within the individual such as “abilities, functioning, or capacities” and statistically “accounts for the relation between treatment and outcome” (Johansson and HØglend, 2007, p. 2; Kazdin, 2007). Mediating variables occupy differing “temporal and causal positions” (p. 2) as well as the “mode of operation (direct or indirect)” (Kazdin and Nock, 2003, p. 1118) all of which require consideration, measurement, and correlation with the outcomes. In contrast to the physical sciences, the numerous idiosyncratic variables and intangible dynamic processes in psychotherapy make it challenging and perhaps counterproductive to isolate singular cause and effect relationship between process and outcome (Hayes et al., 2007; Petrik and Cronin, 2014).

In reviewing what are considered to be mechanisms of change within the psychotherapy literature there is general agreement that aspects of the therapeutic relationship, elements of self-expression, increased levels of consciousness and memory, dialectical tensions, destabilization, ruptures and resolutions, reconfigured and re-storied self-narratives, and self-reflection act as interactive agents of change (Ogden, 1992; Knill, 2005; Hayes et al., 2007; Israelstam, 2007; Zittoun, 2011; Caddy et al., 2012; Forster et al., 2014; Van Lith, 2015; Haas-Cohen and Clyde Findlay, 2015; Lane et al., 2015; Czamanski-Cohen and Weihs, 2016). Additionally, there are numerous theories that identify multiple neurological, psychological, cultural, social, temporal, and intersubjective factors that moderate and mediate the transformation of thought, perception, emotion, and behavior in psychotherapy within and between these identified mechanisms (Bollas, 2002; Hayes et al., 2007; Israelstam, 2007; Harris-Williams, 2010; Brown, 2011; Zittoun, 2011; Forster et al., 2014; Haas-Cohen and Clyde Findlay, 2015; Czamanski-Cohen and Weihs, 2016).

The research in the creative arts therapies related to mechanisms of change is limited in scope and methodology although there are some formative related to mechanisms or phenomena of change. These preliminary theories, in many cases, intersect with those of psychotherapy, suggesting that change occurs within an emotionally attuned therapeutic relationship in which individuals can express themselves through the arts, access and revive memories through sensory and embodied knowledge, gain a sense of safety and relief from tension, reflect and learn about themselves through the therapist/client/arts triadic dialog, progress incrementally through developmental stages, transcend their mental suffering, and enhance their overall psychological and social well-being (Hayes et al., 2007; Israelstam, 2007; Zittoun, 2011; Caddy et al., 2012; Forster et al., 2014; Haas-Cohen and Clyde Findlay, 2015; Lane et al., 2015; Van Lith, 2015; Czamanski-Cohen and Weihs, 2016). Additionally, self-reflection, enhanced levels of consciousness, the necessity for tension, rupture and resolution within a “dialectically attuned” (Israelstam, 2007) therapeutic relationship and the resultant re-imagining and re-creation of personal narratives are all constructs that intersect with the emergent mechanisms of change in the creative arts therapies.

Although creative arts therapies processes associated with transformation may be congruent with those of psychotherapy, differences may be noted in the primacy and value assigned to certain transformational processes associated with the arts-based relational epistemic. For instance in the creative arts therapies the sensory/embodied experiences and relational attunement, the transcendent qualities of imagination and creativity, the reenactment of relational histories within the therapeutic relationship, and the communicative, dialogic, and metaphoric qualities of the arts may assume primacy (Patterson et al., 2011; Gerber et al., 2012; Haas-Cohen and Clyde Findlay, 2015; Van Lith, 2015; Czamanski-Cohen and Weihs, 2016).

The literature reviewed herein reflects promising emergent trends in identifying mechanisms of change in psychotherapy and the creative arts therapies, however, additional exploration is required to advance our knowledge and establish an epistemically sound evidence base for assessing change as it exists and operates specifically within the creative arts therapies. In this paper we aim to add to the emerging bodies of knowledge about proposed and emergent mechanisms and phenomena of change in the creative arts therapies.

## Materials and Methods

The methods for this project included data generation and data analysis phases. These phases included the generation of the records reviewed for the study, the selection of the retrospective study records for coding and analysis, the human subjects ethical institutional board review and approval, the development of the deductive coding system, the organization, coding and categorization of the data, inter-coder alignment, analysis and interpretation, the identification of the primary and modifying themes, synthesis, and presentation of the findings.

In the first part of this section we describe the laboratory context and methods from which the textual data in the study records were generated and, in the second section we present the procedures for our deductive and interpretive coding and thematic analysis of the retrospective data.

### Data Generation

The first phase of our investigation was designed to explore questions related to the nature of therapeutic processes and phenomena of change in the creative arts therapies. To address these questions, we developed a creative arts therapies laboratory course designed specifically to simulate and study arts-based, expressive, and intersubjective phenomena parallel to those in the creative arts therapies. The laboratory course ran for four academic quarters or 1 year over a period of 8 years during which we engaged doctoral students in the Ph.D. in Creative Arts Therapies program, from the disciplines of art therapy, dance/movement therapy, and music therapy, as participant/researchers. We used a method called *Intrinsic Arts-Based Research. Intrinsic Arts-based Research* originates from a psychoanalytic perspective in which the authentic intra- and inter-psychic experiences and data emerge organically through free associative processes within a relational context. In this method, we used and documented our individual and collective intrinsic aesthetic intersubjective experiences as participant/researchers in order to identify and describe the arts-based intersubjective processes that contribute to self/other awareness and narratives, metaphoric expression, insight, and transformation in the creative arts therapies.

The structure of the laboratory experience included a 30-min check-in about afterthoughts and remote reflections from the previous class and discussion of the assigned readings. The second part of the class was 1 h of undirected arts-based exploration in which the students became the participants immersed in all aspects of the intrinsic intersubjective arts-based experience. The goal was to study the authentic experience of the participants as they transitioned in and out of the intersubjective arts-space, experienced the challenges of creating arts-based responses within the intersubjective space. Following this 1 h of authentic intersubjective arts-based exploration, students were asked to step out of their participant role and step into a researcher role devoting 30 min to reflecting upon and documenting their arts-based intersubjective experiences in their journal. Finally, the last portion of the laboratory is a discussion sharing the individual and collective arts-based and intersubjective experiences with the group. Our investigation was designed to answer the following question:

“What are the factors that contribute to therapeutic mechanisms, psychological understanding, meaning making, and transformation within the intersubjective arts therapies process?”

From this simulated creative arts therapies experience students generated multiple types of data which included the arts-based immersive responses, reflective journal entries, group discussions, iterative arts-based reflective responses, and relevant literature. At critical points in the laboratory courses the students would organize, analyze and synthesize these multiple data types through a hybrid of thematic qualitative and arts-based approaches. The results of these analyses were written, arts-based, and performative culminating projects representing the formative findings from each course and summative findings from cumulative courses. These culminating projects became the retrospective records for this research project used to study the mechanisms of transformation.

### Participants

In this research project the “participants” were eight de-identified study records from four students who participated in the laboratory course. The study records were the culminating written and arts based projects representing an analysis and synthesis of the intrinsic arts-based, observational, and reflective data collected by the student participant/researchers at the conclusion of each academic quarter in the laboratory course. Although the course has been in existence for 8 years, the records studied were selected for this study from the year 2012–2016 and represented three different student cohorts. The years from 2012 to 2016 were selected in order to include papers written only by students who had completed the course to avoid potential conflicts related to study participation and course evaluation. The laboratory course was conducted over a period of four academic quarters or 10 months per year, thus, in order to explore the progression of thematic trends over time, we selected one paper from the introductory course and one from the advanced course from each student in each cohort. These records were selected randomly, de-identified, given a participant identification number to replace the name, and paired by course and student. This initial sampling de-identification and pairing was conducted solely by the course instructor/primary author to protect the confidentiality of the students during analysis and publication. The study records and their content were used as primarily aggregate data for thematic analysis with the exception of exemplary de-identified excerpts used to amplify the meaning of the thematic results.

We complied with all human subjects ethical guidelines and had the study approved by the Drexel University Institutional Review Board which is the official human subjects research ethics body in the university. In compliance with the human subjects’ ethical guidelines and with respect for the students and graduates of the program who might have records in the project we notified them about the intention of the investigators to use de-identified aggregate and excerpted data from the records in the study and gave them the opportunity to withdraw their records or review their own records for identifiers. One complication with the de-identification, is that the arts-based investigative responses, central to the intrinsic arts-based research process and the culminating projects, had to be excluded, but descriptions of these processes are still very present in the textual data.

### Data Organization and Coding

The coding system is a deductive or theoretical qualitative research approach designed to arrive at the identification of patterns of evidence and predominant themes relevant to our topic and research questions. The “ ‘theoretical’ thematic analysis would tend to be driven by the researcher’s theoretical or analytic interest in the area, and is thus more explicitly analyst driven” (Braun and Clarke, 2006, p. 84).

We intentionally selected this deductive method for our data analysis to juxtapose and align the extrinsic empirical and theoretical data alongside the inductive data generated in the intrinsic arts based research phase (Braun and Clarke, 2006). Selecting and aligning these two data types and sources was a strategic decision designed to systematically compare, contrast, and integrate the intrinsic and extrinsic perspectives related to transformation for purposes of credibility and authenticity.

To develop the deductive coding system, we conducted a search of the current psychotherapy and creative arts therapies literature from which we identified and extracted the references to mechanisms or phenomena of change and transformation most frequently and consensually reported. We also included emergent evidence based constructs from the course objectives and processes. From these phenomena, we constructed our *a priori* parent coding categories. The *a prior*i parent coding categories were then further modified and defined by child codes that contributed to identifying and modifying specific aspects or operations of the parent codes. Our *a priori* coding categories were organized into the following parent categories for the initial deductive thematic analysis: arts making processes and arts-based research, expression and communication, reflection and awareness, relationships, ruptures, intersubjectivity, and transformation. The child codes and their relationship to their parent codes are presented in Table 1. A coding book including categories and definitions for parent and child codes was developed provided for the coders to enhance inter-coder alignment.

**Table 1:** *A priori* parent and child coding categories.

Parent category code	Child categories
Expression/communication	(1) Free association (2) Resistances (3) Resistances to arts process (4) Metaphors and symbolism (5) Writing (6) Artistic expression (7) Discussion (8) Artistic reflective response (9) Artistic immersive response (10) Emotional expression
Art making processes and arts based research	(1) Transitions (2) Self-consciousness (3) Time (4) Rituals or transporters (5) Creation/free association ***(6) Medium, mode and methods (7) Imagination: flow/transcendence (8) Intersubjective transcendence (9) Sensory/kinesthetic/embodied*** (10) Emotional/affective (11) Cognitive/symbolic (12) Tensions/frustrations (13) Representation
Reflection and awareness	(1) Non-arts-based immersive reflection (2) Memory (3) Remote reflection (4) Artistic reflective response (5) Artistic immersive reflective response (6) Insight/new learning
Relationship	***(1) Attunement/alignment*** (2) Relational misalignment ***(3) Tension/dialectics*** (4) Emotional holding (5) Transference
Ruptures	***(1) Dialectical ruptures (2) Imagination ruptures*** (3) Time and familiarity ruptures ***(4) Relational ruptures*** (5) Dynamic disruption (6) Artistic disruption
Intersubjectivity	(1) Familiarity (2) Unfamiliarity (3) Relationship building (4)‘Narrative **(5) *Intersubjective transcendence*** (cross referenced in art-making processes)
Transformation	*(1)* ***Dialectical rupture and resolution*** (2) Intersubjective witnessing and observation (3) Memory reactivation and emotional reintegration (4) Self-expression, self-discovery and enhanced connection to self (5) Re-imagining, metaphor, and re-storying

*Bold entries represent preliminary thematic results emergent from a priori codes.*

### Defining Coding Categories

The family of parent and child codes were defined not only to identify current trends in the literature, but also to increase inter-coder alignment across the three coders. Each parent code category housed modifying child categories that contributed to the defining properties of the parent category. The child categories were explicitly used in the actual coding process with implicit connections to the parent categories as illustrated in Table 1. Throughout the initial coding process it became apparent, as is the case with most qualitative research coding and analysis, that certain *a priori* codes were assigned more frequently to excerpts in the textual data while others were not used frequently or at all. The most frequently used and meaningful codes emerged as our preliminary thematic results and are highlighted in Table 1 in bold italics.

### Parent Categories

The parent code definitions are included below but space restrictions prohibit the definitions of the child codes here.

#### Expression/Communication

Methods and modes by which thoughts are made visible or audible within an intersubjective context. Examples might be a sensations, embodiment, and emotions expressed through arts, talking, writing, enacting and discussion that releases tension enhances functionality (Zittoun, 2011; Van Lith, 2015; Czamanski-Cohen and Weihs, 2016).

#### Art Making Processes

The process of letting meaning emerge through a dynamic relationship between participants and the art media representing historical and current relational phenomena. Creative activity of making thoughts visible through arts process stimulates complex mind/body interactions contributing to the growth of new neural networks (Zittoun, 2011; Caddy et al., 2012; Haas-Cohen and Clyde Findlay, 2015; Czamanski-Cohen and Weihs, 2016).

#### Reflection and Awareness

Making thoughts visible and learning how to think about and re-think about them through mentalization (Forster et al., 2014) and/or visualization within the presence of another (Zittoun, 2011; Forster et al., 2014). Surrendering to the unconscious, emergent thoughts, sensations, emotions engaging in implicit to explicit processing (Czamanski-Cohen and Weihs, 2016). Creative reflection in the potential space leading to new knowledge and transformation through engagement with and resolution of existential dialectical tensions (Bollas, 2002; Israelstam, 2007).

#### Relationship

An attentive and attuned relational alliance, merging past and present intersubjective narratives, constructed within an emotionally safe space for purposes of facilitating self-expression, self-exploration, reflection, and change. The therapeutic alliance makes room for free talking pre-verbal cognitions, attunement and the emotional space to hold dialectical tensions in the potential space. The potential space allows the individual to: (1) “hear from “his/her “own unconscious”; (2) engage in creative dialectical discourse between me and not-me: and, (3) make the “invisible psychic apparatus of the mind become visible and new narratives to emerge” (Symington, 1996; Bollas, 2002, p. 10; Knill, 2005; Israelstam, 2007; Kazdin, 2007; Brown, 2011; Zittoun, 2011; Forster et al., 2014).

#### Intersubjectivity

Joining with others in the unconscious or conscious co-creation of personal and collective narratives. The co-creation of the group narrative based on the sensory, kinesthetic, emotional, embodied and symbolic forms of knowledge. Awareness and relevance of the presence of others, both peers and leaders, and how this awareness informs and appears in the arts process and product (Knill, 2005; Stern, 2005; Brown, 2011; Zittoun, 2011; Schwartz, 2012; Czamanski-Cohen and Weihs, 2016).

#### Ruptures

Ruptures include mind/body interactions and ways of thinking that interrupt or rupture repetition compulsion, ritual, beliefs, or routine changing meaning and creating new neural pathways (Hayes et al., 2007; Israelstam, 2007; Zittoun, 2011; Forster et al., 2014; Czamanski-Cohen and Weihs, 2016).

#### Transformation

“A significant reconfiguration of perception and thought resulting in the lessening of psychic restraint and pain allowing for the emergence of new psychological perspectives that contribute to living a more creative life” (Gerber et al., 2012, p. 45). Arousal of memories, re-activation of emotions, levels of consciousness resulting in new learning and insight (Hayes et al., 2007; Israelstam, 2007; Zittoun, 2011; Van Lith, 2015; Lane et al., 2015; Czamanski-Cohen and Weihs, 2016).

### Coders and Inter-Coder Alignment

The coders included one alumnus and one current PhD Candidate in addition to the laboratory course instructor. All students and alumni who had participated in and completed the laboratory course were invited to participate in the project. Each student coder had taken the course at a different time and with a different cohort while the instructor had been present for all of the courses. As a result each coder brought a different perspective based upon his/her experiences and roles as participant/researcher in the course factoring into and enriching the assignation and interpretation of the codes. We coded in pairs for each record in attempts to contribute to the credibility of the results by including multiple perspectives and member checks.

Based upon these multiple perspectives we recognized the need to evaluate the inter-coder alignment. Evaluation of the inter-coder alignment occurred in two ways. First, the coders met periodically throughout the coding process to discuss the inter-coder convergences and divergences of the code assignations. Second, we used the analytic functions of the Dedoose cross-platform application which allowed us to view the distribution, frequency, and co-occurrence of codes across coders.

### Coding Procedures and Data Analysis

Our procedure for coding and analyzing the data from the eight study records included: (a) importing the *a priori* codes, definitions, and written texts into a cross-platform application called Dedoose; (b) employing the services of three coders; (c) immersion in the textual data and code assignation process; (d) inter-coder alignment checks; (e) analysis for thematic predominance and “keyness”; (f) interpretation and synthesis (Braun and Clarke, 2006). Initially, we used a semantic method to code excerpts for literal content based upon the definitions for the *a priori* parent and child categories. “With a semantic approach, the themes are identified within the explicit or surface meanings of the data, and the analyst is not looking for anything beyond what a participant has said or what has been written” (Braun and Clarke, 2006, p. 84).

During the semantic analysis we identified the emergence of predominant categorical patterns of evidence from the *a priori* parent and child categories. We used Dedoose to explore the patterns of evidence and most frequently coded categories. The most frequently coded categories (Table 2) were then organized and aggregated with their *a priori* definitions. We then reviewed and organized the textual excerpts, explored the thematic content of the excerpts, examined co-occurrences of codes and inter-coder alignment, and then re-organized, revised, and collapsed the categories into new but related categories which became or data sets. These data sets were created according to the frequency and contextual predominance, co-occurrences, and textual meaning, resulting re-interpreted and integrated categories representing a merger of the extrinsic and intrinsic data.

**Table 2:** Preliminary data sets with *a priori* definitions.

Parent code	Child code
Arts-based expression	**Art making processes medium, mode and methods:** The method or methods of artistic expression as change agents (Knill, 2005; Van Lith, 2015)
Art making processes and arts based research	**Art making processes imagination flow/transcendence:** Engagement in artistic levels of consciousness that facilitate imagination, transcendence of time and thought rigidity patterns (Caddy et al., 2012); transcendence beyond physical and mental strife or illness (Van Lith, 2015); restoration of play to expand perception of possibilities (Knill, 2005); “desired level of consciousness attained once fully engaged in the arts process… beyond confines of physical world while allowing peripheral awareness of it…” promotes meditation, introspection, reflection, and empathy (Gerber et al., 2012, p. 44)
	**Art making processes intersubjective transcendence:** State of consciousness using imagination to surpass the physical boundaries of bodily separateness allowing for the imagining and understanding of and empathy for the other (Bollas, 2002; Gerber et al., 2012). The arts are facilitators and mediators of these dynamic changes due to the inherent intersubjective nature of the arts in which the engagement with self/other or self/object is an ongoing and enlightening dialectic process of discovery (Hagman, 2005).
	**Art making processes sensory/kinesthetic/embodied:** Expression of sensory preverbal embodied forms of knowledge, artifacts and memory stimulating emotional systems in the brain – without the assignation of language (Bollas, 2002; Chilton et al., 2015; Czamanski-Cohen and Weihs, 2016)
Relationship	**Relationship tension and dialectics:** Tension, frustration, at critical points of ambiguity in the process that have the potential to result in creative transformation or collapse. The dialectic between the “me and the not me” creates corollary existential and psychological life/death experiences resulting in the tension necessary for reflection, new insight, and creative transformation (Israelstam, 2007; Zittoun, 2011)
	**Relationship attunement and alignment:** Emotional attunement to and alliance with the other’s emotional life and invisible psychic apparatus (Bollas, 2002) “… to facilitate the opening of a creative reflective space in which positive transformation can occur” (Israelstam, 2007, p. 592); therapist’s surrendering to his/her own unconscious can allow one to “catch the drift” of the patient –“unconscious communication” (Bollas, 2002, p. 12)
Ruptures	**Ruptures imaginational ruptures:** Ruptures caused by imagination –sensory, embodied, emotional psychic processes that evoke memory and fantasy require relinquishment of control, embrace the anxiety and trust in the imaginative process. Transcends and interrupts rigid modes of thought through accessing right-brain functions (Bollas,2002; Knill, 2005; Czamanski-Cohen and Weihs, 2016)
	**Ruptures relational ruptures:** Ruptures in the relationship due to anxiety, fantasies, tension or breakdown in the relationship resulting in potential change (Knill, 2005; Israelstam, 2007; Gerber et al., 2012; Forster et al., 2014); ruptures and resolution moving through “acknowledgment through understanding and assimilating warded off feelings to closure” (Bennet et al. as cited in Forster et al., 2014, p. 8)
Transformation	**Transformation dialectical rupture and resolution:** New learning and insight from resolution of dialectical ruptures meaning on the creative edge between the drive for newness and clinging to the old and familiar; systems of thought and experience are destabilized and re-stabilized through the reconstruction of new perceptions (Lynch et al., 2006; Hayes et al., 2007; Israelstam, 2007; Zittoun, 2011; Forster et al., 2014).
	**Dialectical Ruptures:** Tensions from self-narrative contradictions, internal/external dialogs at critical points in the process resulting in ruptures and re-constructed linguistic structures, resolutions, and meanings (Israelstam, 2007; Zittoun, 2011; Forster et al., 2014)

**FIGURE 1:**
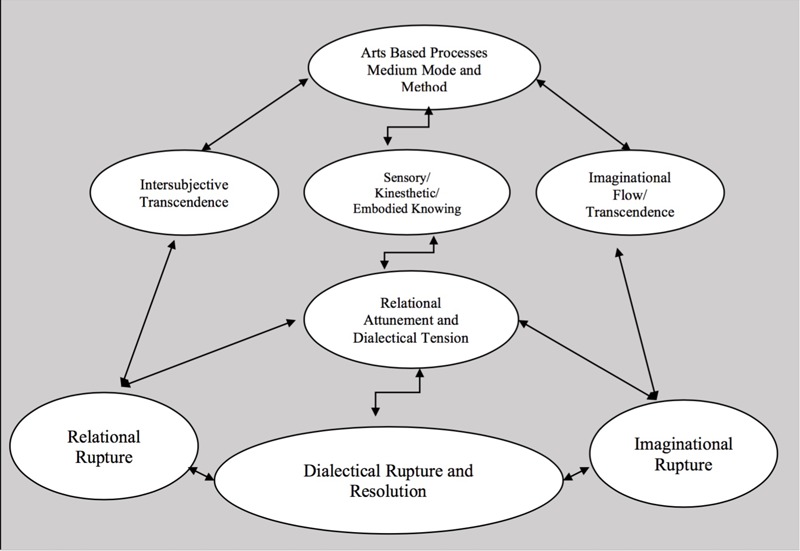
Kinetic mobile system for dynamic change.

**FIGURE 2:**
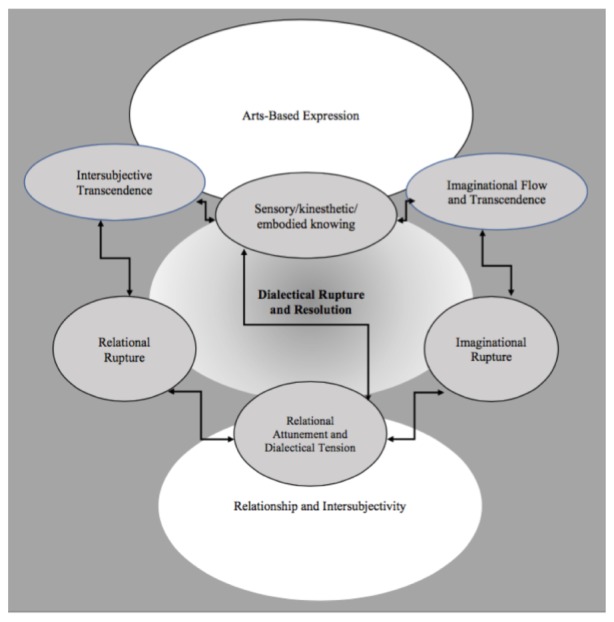
Figure/ground system for dynamic change.

**FIGURE 3:**
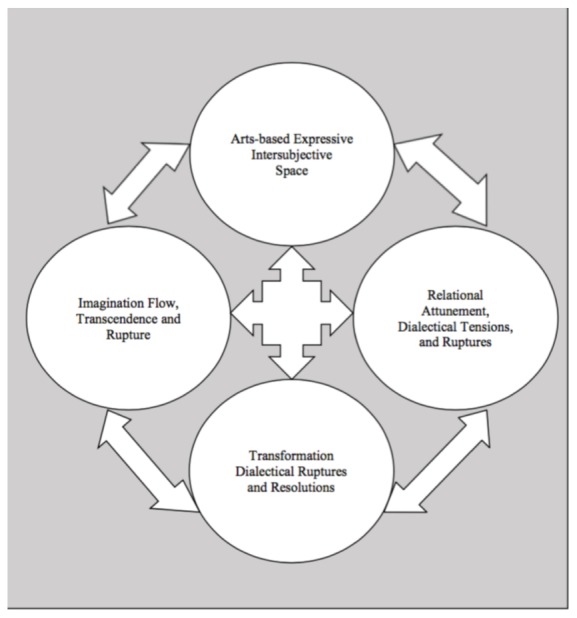
Orbital system for dynamic change.

**FIGURE 4:**
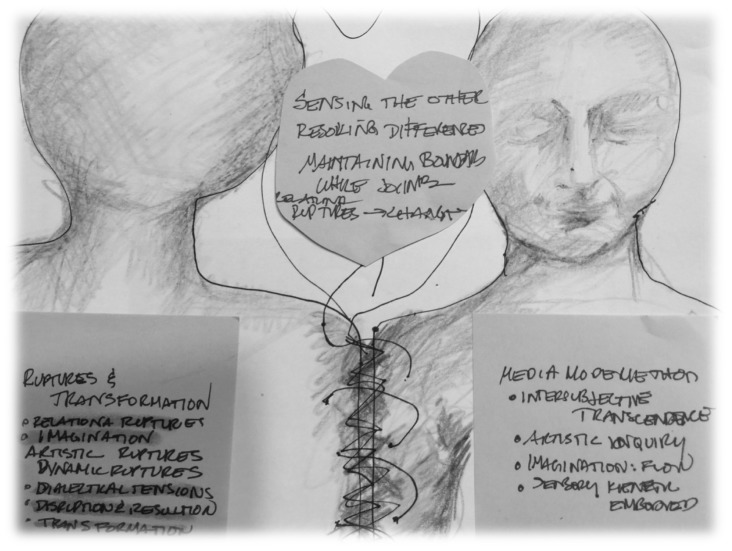
Art-based thematic initial conceptualization—dialectical rupture and resolution. Original in color. Created by primary author.

From this point, it was natural to move from a semantic analysis into more interpretive work by exploring the latent content (Braun and Clarke, 2006). Within this final interpretive phase we first re-named these new integrated data sets and their meanings which became the three primary themes and the related modifying and defining themes. Then we focused on exploring the relationship between the primary and modifying themes relative to the phenomenon of transformation. We used some arts-based methods (Figure 4) and diagrams (Figures 1–3) for the purposes of conceptualization, visualization, interpretation, and thematic synthesis. In exploring the relationships within and between the thematic constructs, we created dynamic interactive systems of change comprised of these transformative thematic phenomena (Figures 1–3).

## Results

The results of our preliminary analysis yielded the identification of three primary themes modified and defined by interactive sub-themes related to transformational phenomena in the creative arts therapies. In this section we present the themes, define and describe each theme, present exemplars to amplify the meaning, and provide a summary synthesis of the thematic results as related to our research questions. As we present these results we re-emphasize the limitations of this analysis. The limitations became more apparent as we embarked on this project and realized that the scope, depth and breadth of our data might extend beyond the time and space constraints for this article. Our assessment of the data at this point in our analysis is that they are extremely rich and meaningful holding multiple implications for further research, theory building, and practice therefore requiring additional analysis.

Within those limitations, we identified several preliminary thematic patterns of evidence that were distributed throughout and across five of the original *a prior*i parent categories of *arts-making processes and arts-based research, relationship, ruptures, intersubjectivity, and transformation*. Within those predominant parent categories, the child categories or sub-themes that emerged included *medium, mode and method, imagination flow and transcendence, intersubjective transcendence, sensory, kinesthetic embodied knowing, attunement/alignment, tension and dialectics, imaginal ruptures, relational ruptures, and dialectical rupture and resolution.* These categories achieved primacy through both the frequency of occurrence and the relevant meaning or “keyness” to the inquiry. The “…‘keyness’ of a theme is not necessarily dependent on quantifiable measures, but rather on whether it captures something important in relation to the overall research question” (Braun and Clarke, 2006, p. 82).

The key categories were aligned with the original definitions (Table 2) and then these categories and their excerpts, selected across participants based upon their “keyness” to the inquiry, were analyzed for intersecting meanings, re-arranged and collapsed into data sets to form new integrated categories. These new integrated categories and their meanings were re-organized, collapsed, and rearranged to become the three primary themes and the related modifying and defining themes.

### Primary Themes and Modifying Sub-Themes

Through our analysis we identified the following primary thematic phenomena along with their modifying and defining thematic constructs.

(1) Rupture, Resolution, and Transformation: Dialectical Rupture and Resolution, Relational Ruptures and Imaginational Ruptures;
(2) Relationship and Intersubjectivity: Relational Attunement, Dialectical tensions, Intersubjective transcendence
(3) Arts-based expression: Imaginational Flow/transcendence, sensory/kinesthetic/embodied levels of knowing, and intersubjective transcendence, medium mode and method.

#### Ruptures, Resolutions, and Transformation

Transformation is a major category and central focus for this study. The definition for transformation was the arousal of memories, re-activation of emotions, and levels of consciousness that mediate the new learning and insight through dialectical rupture and resolution (Hayes et al., 2007; Israelstam, 2007; Zittoun, 2011; Lane et al., 2015; Van Lith, 2015; Czamanski-Cohen and Weihs, 2016). Furthermore transformation includes a “… significant reconfiguration of perception and thought resulting in the lessening of psychic restraint and pain allowing for the emergence of new psychological perspectives that contribute to living a more creative life” (Gerber et al., 2012, p. 45). This theme includes the most frequently cited category of dialectical rupture and resolution along with inter-related defining constructs of relational and imaginational ruptures.

Dialectical rupture and resolution was the most frequently coded defining theme describing key transformative actions and moments. This theme increased in the frequency of coding over time during the laboratory course (Table 3).

**Table 3:** Progressive thematic coding frequency over time.

Data sets/thematic phenomena	716	719
Imagination: flow	10	20
Intersubjective transcendence	9	20
Medium, mode and methods	23	24
Sensory/kinesthetic/embodied level	21	14
Attunement/alliance	11	20
Tension/dialectics	4	14
Imagination ruptures	4	10
Relational ruptures	7	16
Dialectical rupture and resolution	7	62

*716 = Introductory Course; 719 = Advanced Course.*

Dialectical rupture and resolution is inter-connected to multiple dynamic processes including relational attunement, imaginational flow and transcendence, and intersubjective transcendence and their dialectical counterparts of imaginational and relational ruptures. The dialectic between these relational and imaginational attunements, flow, and ruptures represents contradictions and tensions between the drive for progressive innovation and discovery and the longing for familiar recalcitrance–the known and the unknown. These tensions create the conditions for a system of dynamic change through destabilization and de-construction, reflection, re-construction, and re-stabilization resulting in insight, illumination, psychic growth, and new personal and intersubjective narratives (Israelstam, 2007; Zittoun, 2011; Forster et al., 2014).

##### Excerpts dialectical rupture and resolution

Ebbing and Flowing” addresses the natural fluctuations of life. Nothing stays the same – there is a constant flux. In our studio class we became aware of such dichotomies as death and life, distance and closeness, divorce and intimacy, facing a threat and running away from it.
In an attempt to understand my inherent tensions between consonance and dissonance, my ritual of staying in my learned comfort zone and the spontaneity of newness, and my holding onto of the familiar while letting go and growing, I wrote the following musical lyric:
  Built up rattled nerves
  Lay them flat on the ground, breath the sound in of dissonance
  Buzzing flies around your ears
  Play beginnings of life, stir the pot, pull the freedom near
It was through this lyrical writing where I helped myself reflect on my tension, embrace the different emotions I was feeling, and come to terms with this tension. I found that through the course, even though this dialectic existed and challenged me, I was more accepting of it as time passed. I understood that this dialectic would become present and the task would simply be adapting around or within it.
However, when we arrive at communal art making, we can face terrors, loss, and trauma and not be broken. We can experience sadness and anger and not fall down. Creating art together allows us to cope with the darkness, make meaning of our experiences, transform our existence, and find hope and peace. Through art we find resilience.
Relational ruptures are inextricably connected to the dynamic between relational attunement and dialectical tensions. The frequency of coding in this category increased over time during the laboratory course (Table 3).
Ruptures in the relationship are due to anxiety, fantasies about self/other, internal/external dialogs, and/or the breakdown in the relationship from disappointment and realization (Knill, 2005; Israelstam, 2007; Gerber et al., 2012; Forster et al., 2014). The successful resolution of these ruptures necessarily occurs within an emotionally held or “dialectically attuned” (Israelstam, 2007, p. 592) relationship in which creativity is used to re-imagine, explore and resolve the dialectical relational tensions. This process is iterative, hopefully progressive and transformative, but exists along a precarious dialectical edge the navigation of which can lead to either creativity and illumination or destruction and devastation (Israelstam, 2007, p. 592).

##### Excerpts relational rupture

About half way into the class, we were both on the floor, one of my classmates began to tear up and appear visibly upset. At that time I was tending to myself and my own needs, calmly breathing, humming a little melody, and overall in a peaceful state. Her condition aroused an immediate response from me, first one of surprise and helplessness, followed by one to breathe and attune to her. I recognized her stooped position and the passive weight in her body. Suddenly, seemingly out of nowhere, a lightness overcame me and my hand approached hers with a playful, non-threatening movement. She responded and we were engaged in a time of short play…
The sunken and hollow body position in my peer (Day 2) confounded me and, while I slowed down my movements and attuned to her, it wasn’t until after everyone had worked their way through sadness that it finally hit me. It was inevitable that it would affect me but the way it did, superficially at first (possibly defending myself by setting boundaries?) with an intense delayed sensation of it, was unexpected.
The spiral of intersubjective relationships among the participants, and the participants’ use of space, represents dynamic changes. We are always positioning ourselves in relation to each other and always sensing where we are and how we are. For example “when we feel open and receptive, we tend to move toward others and reduce or dissolve our physical boundaries” or in comparison “when we feel threatened or in conflict, or there is no trust yet built, we retreat from others and shore up our physical boundaries against them” (Dosamantes, 1992, p. 9).
The triangle formation appeared while writing a song and moving in response to the lyrics, as a song and dance gave the participants a creative vehicle for representing the conflict that had appeared between the student-participants and their instructors.
An understanding and empathy for what others in the class were feeling existed, even if their feelings were in opposition of my own. This made it clear that our feelings were on a dialectic continuum within an intersubjective context.
Imaginational ruptures are dialectically related to imaginational flow and transcendence. These themes progressively increased in coding frequency over time during the laboratory course (Table 3).
Ruptures in imagination include sensory, embodied, emotional psychic processes that evoke memory and fantasy, cause disruption in states of consciousness, and flow, and collisions between fantasy and reality. Imaginational ruptures are transitions in levels of consciousness requiring relinquishment of control, suspension of familiarity, renouncement of mundanity, and interruptions of rigid modes of thought (Bollas, 2002; Knill, 2005; Czamanski-Cohen and Weihs, 2016). Transitioning into the world of imagination is a dialectical and dynamic process creating tension between the real and imagined, the present and absent, and the known and unknown resulting in a conflict and resistance to the process (Knill, 2005; Israelstam, 2007) and negotiation of a creative resolution. Imaginational ruptures and resolutions are considered to be central to achieving states of flow, transcendence, progressive and creative transformation necessary for insight and growth.

##### Excerpts imaginational ruptures

The artwork that I created in the initial classes represented the unknown, the muck. The ideas that formed were abstract and unclear. The images from these first classes were of the free flowing ink, and the muck, and the discussion that followed the classes reflected that other group members had a similar experience. It was an important stage because by allowing to freely explore the artistic media, clear symbols started to emerge. It was from the muck, if you wish, that the symbols of the tree and the bird grew.
As the heaviness lifted in the room, a Blues rhythm picked up and we all…engaged in a time of rhythmic movement and music making. I thoroughly enjoyed this and thought I could go on enjoying it when, unexpectedly, I no longer did.
An image of an incoming storm mirrored that experience for [Participant 1]. She wrote in her journal: “I can feel something coming up, taking form. The air is thick with anticipation of a storm. I feel like something is going to happen, resolve, open up, come together. What it is? I don’t know. How? I don’t know. But I can sense a certain tension and an anticipation of something.”
…Observed that while both music and movement evoked a response, they seemed to latch on to different facets of our emotions. This was most noticeable during my moving to Schubert’s “die liebe Farbe,” when movement allowed me to gain an auxiliary dimension of hurt, adding components of confusion and fragmentation.

#### Relationship and Intersubjectivity

Relationship and intersubjectivity includes the modifying and defining themes of relational attunement and dialectical tensions and intersubjective transcendence (cross referenced in the arts based expressive process theme). These themes progressively increased in coding frequency over time during the laboratory course (Table 3).

This theme refers to the attunement to others at the most fundamental emotional and unconscious level and joining in the co-creating, re-imagining, and transforming our personal and intersubjective narratives. The construction of these narratives includes the dialectic between various levels of trust/mistrust, distance and closeness, intimacy and alienation necessary for attunement to the most authentic, emotional, and fundamental of human experience and connection (Knill, 2005; Stern, 2005; Brown, 2011; Zittoun, 2011; Schwartz, 2012).

Relational attunement and dialectical tensions emerge and co-exist in the intersubjective arts-based expressive experience and in combination are akin to relational ruptures. Relational attunement and dialectical tensions both increased in coding frequency over time during the laboratory course (Table 3).
Relational attunement includes alignment to the other’s emotional life and invisible psychic apparatus (Bollas, 2002) using imagination to facilitate “… the opening of a creative reflective space in which positive transformation can occur” (Israelstam, 2007, p. 592). Relational dialectical tensions refer to the dialog between alignment and misalignment, the “me and the not me” creating corollary existential and psychological life/death experiences resulting in the tension necessary for rupture, reflection, new narratives and insight, and creative transformation. The combination of attunement and dialectical tension occurring within the potential space appears to be essential to the construction of authentic relational knowing and attachment.

##### Excerpts relational attunement

When we create art, all differences melt and become irrelevant. We come together and connect through art. Sometimes coming together may take a while, other times it seems effortless broadly define consonance not just in relation to musical terms, but with relevance to structure, aesthetic appeal, and a person’s inherent, natural tendency.
I tried to anticipate X and Y’s rhythms and movements, trying to stay connected through cognitive awareness…I became part of the movement, the rhythm. I followed, I lead, I existed, interconnected to sounds and feels and raw emotion. It was exhilarating and so calmingly beautiful in the same space.
… seeing an expressive movement, mirroring its essence and feeling a sensation; experiencing an emotion, then moving the body in congruence with it; hearing a musical piece, adjusting the movement to the nature of the music and having an emotional response. In short, the interrelatedness of movement and emotion was present throughout, no matter what initiated what.

##### Excerpt relationship dialectical tensions

In the same artistic experience, one of us could feel comfort and another could feel discomfort, and we somehow transitioned within and around this space as individuals as well as a group within the experience.
Alternately, dissonance is defined in opposition to these terms, being disorganized, different, and disconnected. Within the consonance and dissonance themes were subthemes of ritual and spontaneity (a dialectical term discussed in Israelstam’s, 2007 article), holding and growing, sameness and difference, and connected and disconnected.”
“The mutual awareness of agreement or disagreement and even the realization of such understanding or misunderstanding” (Gillespie and Cornish, 2010, p. 19).
What happened with me when I was moving, that I was reminded of two different types of responses to other people, also informed me of proxemics. It is now clear to me that distances between people differ according to relation (close-distant, personal-professional, or first time-know).

#### Arts Based Expression

The theme of arts-based expression represents the process of letting meaning emerge through a dynamic triadic relationship between participant/researcher, media/mode/method and the art making. This theme remained constant in coding frequency across time during the laboratory course (Table 3). The secondary themes in this category are the sensory/kinesthetic and embodied ways of knowing, imaginational flow and transcendence, and intersubjective transcendence.

The arts-based expression requires the immersion in creative process that makes thoughts visible using the media, modes, and methods of artistic expression (Levine, 2005; Zittoun, 2011; Caddy et al., 2012; Haas-Cohen and Clyde Findlay, 2015; Czamanski-Cohen and Weihs, 2016). Immersion in the expressive arts process requires engagement in the dialectic between resistance, rupture, and resolution, surrendering to the imagination, and ultimately entering a transcendent state of consciousness and imaginative flow, acute relational attunement, and empathic intersubjective transcendence.

Sensory/kinesthetic/embodied ways of knowing, are primal unconscious forms of cognition that hold the artifacts of our earliest memories and stimulate emotional systems in the brain without the assignation of language. The arts experience uses sensory/kinesthetic/embodied and imaginal knowledge to transcend time retrieving the primal experience and replicating the original emotional response (Bollas, 2002; Chilton et al., 2015; Czamanski-Cohen and Weihs, 2016). Just as in infancy, due to its primal nature, sensory/kinesthetic/embodied and imaginal knowledge creates acute relational attunement at the most fundamental emotional level. This theme remained constant over time with a slight decrease in the frequency coding over time in the laboratory course (Table 3).

##### Excerpts sensory/kinesthetic/embodied knowing

Initially the ocean drum and the swaying around me took me to a peaceful place, but over time the feeling shifted in the room. The movements slowed down, all DMTs were on the floor. Harmonies sung in a minor key, combined with the restricted movement and contracted body language around me evoked a state of deep, penetrating sadness. I found myself rocking, crying, remembering. This emotional state was hard to shake, even when I made physical changes (standing up, increasing energy).
Finding Resilience through Art” via movement. With my eyes closed there was little coping, however, as I opened them and began to create with my hands, first in a miniscule manner, but over time more and more elaborately, I was able to gain a new perspective and move outside of myself.
When I was dancing I noticed that my body takes different positions and shapes in space. I noticed that my movements varied. Once I was moving slowly, and other times quickly with more expression. Once I was using just parts of my body, in separation, and other times my whole body was moving. There were times that I was in a low position, and there were times that my body took shapes when I was standing or jumping. After a while, I still wasn’t sure what all of this meant to me, or if it had any meaning at all. I decided to move naturally for a while, warming up my body, as in preparation for deeper exploration.
Imaginational flow/transcendence relates the “desired level of consciousness attained once fully engaged in the arts process… [transcendent]beyond confines of physical world while allowing peripheral awareness of it…” promoting meditation, introspection, reflection, and empathy (Gerber et al., 2012, p. 44). Artistic levels of consciousness also refer to a state of imaginational flow bypassing thought rigidity and resulting in the “growth of new neuron networks” (Caddy et al., 2012, p. 328). Surrendering to the imaginational flow through free association and attunement to sensory embodied ways of knowing results in restoration of play, loss of time consciousness, transcendence beyond physical and mental strife, and expansion of the perception of possibility (Knill, 2005; Van Lith, 2015).
This theme increased in coding frequency over time during the laboratory course (Table 3).

##### Excerpts imaginational flow/transcendence

I lay down on a floor stretching my mind to the limit of its extension to find answers. Themes, themes, themes… like I heard this world all over the place, all the time… I felt like I couldn’t find it. I felt stuck. How I am supposed to find it? I closed my eyes and my mind went somewhere far, far away. In my mind I was levitating over the mountains, rivers, seas, oceans and dessert. I felt relaxed and calm. My breath was stable and my heart beat pretty calm. I stayed there for a while, however, I lost control of time. I think I might have fallen asleep as at one point I felt cold, so cold that I curled up in the embryonic positions shaking and tensing my muscles. I still didn’t want to leave the floor it felt so supporting, however, the emerging cold made me move in a very uncomfortable way. I slowly began to twist, bend, writhe with a extremely bound muscle tension and without any direction. Just shaping my body through space and to adapt to the cold. Suddenly I hear this loud and annoying sound BZZZZZ and I stand up on straight legs. It was so unexpected as a quick unexpected frog coming out of a dark sleepy pool. Dark sleepy pool? Unexpected frog? I stopped myself for a while and wondered if I feel ok. Frog, pool, splash, unexpected… Yes! I have an idea.
Intersubjective transcendence describes the levels of consciousness attained using imagination and immersion in arts-based processes to transcend the physical boundaries of interpersonal separateness and enter the sensory, emotional, and imaginal world of “the other” enhancing attunement, understanding, and empathy (Symington, 1996; Bollas, 2002; Gerber et al., 2012). This theme increased in coding frequency over time during the laboratory course (Table 3).

##### Excerpt intersubjective transcendence

Through my movement inquiry I noticed that true togetherness, a connecting of the hands, wiped away all the differences. Togetherness and connection in DMT is promoted through the therapist’s mirroring or reflecting of patients’ movement qualities. This results in an increased degree of somatic and emotional understanding as well as empathy.
At the beginning there was a sense of slowness, careful attention and intimate contact among the participants, and deeper exploration of individual problems, however, expressive movement was limited. Later during the session, expressive movement emerged and There was a sense of meditative and trance dance, in relation to expressive and meditative music.

### Progressive Thematic Coding

In addition to analysis of the data thematically, we also wanted to explore how these thematic results emerged, sustained, developed, or diminished over time in the laboratory course. The progression of the themes over time was tracked by the frequency with which these thematic categories were coded in the study records from the introductory course (716) to the advanced course (719). Interestingly, all of these coded categories except for two, increased in the coded frequency over the progression of the course. Of particular note is the dramatic increase in the frequency that dialectical rupture and resolution was coded along with imaginational flow, relational attunement, relational and imaginational ruptures and tension and dialectics generally doubled in frequency. Medium mode and method in the arts-based expression category and sensory, kinesthetic and embodied knowledge remained the same over time with the latter dipping by just a few instances. Although there are numerous interpretations of this result, the distinct trends bear noting and further investigation (Table 3).

In summary, the primary thematic categories of ruptures, resolution, and transformation, relationship and intersubjectivity, and arts-based expression together with their modifying and defining themes, represent what may be transformative phenomena equivalents to mechanisms of change in the creative arts therapies. Due to the pluralistic intersubjective nature of reality and aesthetic knowledge in the creative arts therapies, these transformative phenomena are conceptualized as interactive dynamic systems of change in contrast to singular, linear, causal mechanisms of change. We have proposed several dynamic systems to illustrate how we envision these thematic phenomena interacting with one another to describe transformation (Figures 1–3) which are discussed in more detail in the Section “Discussion.”

## Discussion

In this preliminary phase of our research study, we have explored formative phenomena that, taken together, may be descriptive of the ways in which change occurs in the creative arts therapies. In this section we explore the dynamic interactive relationships between the primary and modifying themes and propose how these interactive phenomena might form a system of change. We also address the limitations of the study and how those limitations both elucidate the results and illuminate directions for future research. Finally, we recommend methods of evaluating these formative dynamic constructs of change in research, clinical practice, and the development of an evidence base for the creative arts therapies.

In interpreting the findings for this study, it is essential to re-emphasize that these findings represent a small but in depth sampling of data generated by student participant/researchers from a laboratory course simulating the creative arts therapies experience. Therefore, considering the interpretation and transferability of these constructs to theory building and clinical practice resides within and is limited by that context. With that said, we also may have to re-consider the hegemonic criteria, implicit in that statement, by which we typically evaluate research results. For instance, in this study a method for evaluating the results of this study have to more mindfully include a paradigm shift. In this paradigm shift it may be more useful and relevant to select arts-based or qualitative research evaluative criteria that are more aligned with the aesthetic intersubjective mental model (Greene, 2007) or worldview of the creative arts therapies in contrast to a quantitative research reductive mindset more aligned with physical sciences. Within an aesthetic intersubjective mental model, the themes we identified represent phenomena that are dynamically and spatially inter-related presuming change as related to interaction as opposed to singularly static linear and causal constructs. Consequently, we are exploring the construction of meaning and change through kinetics, dynamics, inter-relatedness, and dialectics reflective of the ontological and epistemic nature of our fields and these thematic phenomena. Contextualized within these paradigmatic and methodological shifts we explore the dynamic systems of change created from these thematic phenomena, their implications for clinical and research theory and practice.

### Dynamic Thematic Synthesis

The primary, modifying and defining themes identified in this study represent dynamic phenomena that dialectically adjoin and collide in the arts-based relational context descriptive of qualities of perceptual, emotional, relational, and behavioral experience contributing to change in the creative arts therapies. The primary interactive thematic constructs from our analysis are: (1) ruptures, resolutions, and transformation; (2) relationship and intersubjectivity; and, (3) arts-based expression. These primary thematic constructs are mediated by a dynamic and iterative interaction with the modifying and defining thematic phenomena of *dialectical rupture and resolution, sensory/kinesthetic/embodied knowledge, imaginational flow/transcendence and rupture, relational attunement and dialectical tension, relational rupture and intersubjective transcendence*. The dynamic interaction between these phenomena occurs in an arts-based expressive and intersubjective holding environment that can tolerate, emotionally regulate, and accommodate the creative and relational dialectical processes of contradiction, tension, and resolution necessary to promote change. We explore these dynamic constructs in more depth, examine different interactive configurations, and consider their relevance as a system of arts-based relational mechanisms of change.

Central to our discussion of mechanisms of change is the operational definition we used for transformation which was the arousal of memories, re-activation of emotions, and levels of consciousness that mediate new learning and insight through dialectical rupture and resolution (Hayes et al., 2007; Israelstam, 2007; Zittoun, 2011; Lane et al., 2015; Van Lith, 2015; Czamanski-Cohen and Weihs, 2016). Furthermore, transformation is a “… significant reconfiguration of perception and thought resulting in the lessening of psychic restraint and pain allowing for the emergence of new psychological perspectives that contribute to living a more creative life” (Gerber et al., 2012, p. 45).

Within the literature and our data dialectical rupture and resolution was identified as one of our most predominant and overarching themes instrumental to transformation. Dialectical ruptures and resolutions are the pervasive ongoing and driving forces central to change, fueling creative, relational, and psychological growth from the friction between seeming contradictions in thought, belief, and experience. Typical dialectical tensions emerge from the existential anxieties and conflicts between the drive for progressive innovation and the gravitational longing for familiar recalcitrance–seeking the known from the unknown, creating something from nothing. In our study, the dialectical rupture and resolution process was mediated primarily by the dynamic interaction between relational attunement, imaginational flow, and intersubjective transcendence and their correlates of relational and imaginational ruptures. These tensions create the conditions for a system of dynamic change through iterative phases of destabilization and de-construction of pre-existing beliefs and narratives, re-construction of new narratives, and relationship re-stabilization resulting in new insight and illumination (Israelstam, 2007; Zittoun, 2011; Forster et al., 2014).

Essential to the resolution and reparation of these dialectic tensions and ruptures are the interrelated thematic constructs of relationship and intersubjectivity and arts-based expression. In our study and, in the creative arts therapies and psychotherapy literature, the construction of a relationally attuned, emotionally held and responsive intersubjective culture is deemed essential for facilitation of surrender to and engagement in the arts-based expressive processes. Surrendering to the imagination, necessarily includes engagement in dialectic between resistance, rupture, and resolution ultimately allowing for the attainment of the transcendent state of imaginative flow, acute relational attunement, and intersubjective transcendence. Consequently, the intersubjective arts based expressive process juxtaposes imaginational flow and relational attunements and their correlate dialectic ruptures creating an ongoing transformative dialog necessary for resolution and change –jarring fixed and rigid beliefs that impede progressive expression, conceiving, re- imagining and birthing new systems of thought and perception, contributing to reparation, synthesis and transformation within a strong relational attuned emotionally holding environment.

Implicit in and central to these relational, arts-based, and intersubjective processes, is the invisible and influential role of sensory/kinesthetic/embodied knowledge and relational attunement. Sensory/kinesthetic/embodied modes of knowing and communication, originating from the beginning of life, create relational attunement at the most fundamental, poignant, and penetrating levels inaccessible through more traditional means of communication. Sensory/kinesthetic/embodied knowing within the relational or potential space contributes to the fluctuating levels of consciousness essential for imaginational flow and intersubjective transcendence (Ogden, 1992; Hagman, 2005; Israelstam, 2007). This state of flow facilitates levels of consciousness that transcend the limitations of physical, temporal, and spatial boundaries enhancing interpersonal awareness and empathy and the basis for the construction of authentic emotional relationships that could both withstand and facilitate ongoing ruptures and resolutions.

We propose, therefore, that the dynamic interaction between these thematic phenomena in varying combinations and at varying strategic times, within the therapy and the therapeutic relationship, generates transformative responses. This is a preliminary study with formative qualitative evidence about these transformative phenomena. That evidence combined with the progressive frequency by which our categories were coded across time in the study posits some intriguing ideas and questions. In that finding all but two of the primary and modifying themes increased in the frequency by which they were coded over time (Table 3). Of particular interest is dramatic increase in the frequency of coding for the theme of dialectical rupture and resolution, within the overall theme of ruptures, resolutions and transformation, along with modifying themes of relational and imaginal ruptures. This increase along with the concomitant increases in relational attunement, intersubjective transcendence, and imaginational flow suggests that there is perhaps a dynamic interaction between these experiences that create the progressive conditions necessary for facile engagement in the dialectical rupture and resolution process essential for change. In other words, the interactive mechanisms or phenomena from the study progressively contribute to the creation of a relationally attuned intersubjective culture in which imagination, dialectical tensions, and arts-based expressive process develop over time and indeed might be contribute to change and transformation.

In considering the nature, meaning, relationship, and progression of these preliminary and formative interactive dynamic phenomena we revisit the concept of mechanisms of change. With regard to mechanisms of change and how change occurs in the creative arts therapies, we think that our findings necessitate a paradigm shift from a singular causal action to a dynamic interactive system between multiple human phenomena. In this paradigmatic shift and proposed model, in contrast to more traditional definitions and evaluation of mechanisms of change, the relationship of the mechanism and the outcome is not linear and measureable but rather dynamic, multi-dimensional, and descriptive.

### Mechanisms of Change: Paradigmatic Considerations

A review of the nature and relationship of our thematic findings relative to the extant concepts of mechanisms of change suggests paradigmatic and methodological reconsiderations. Mechanistic research resides predominantly within a post-positivist paradigm in which a statistical and singular causal relationship is created between the mechanism, intervention, and the outcome as the explanation of how change occurs (Kazdin and Nock, 2003; Kazdin, 2007). There are particular bodies of knowledge and domains of scientific research in which this approach is warranted resulting in valuable answers to specific questions. However, due to the nature of reality and forms of knowledge in the creative arts therapies, mechanisms of change may require reconsideration, redefinition, and reconfiguration. As described previously, in the creative arts therapies we deem reality to be pluralistic and intersubjectively co-constructed while the related forms of aesthetic knowledge are necessarily idiosyncratic, circuitous, dialectic, dynamic, and emergent. These basic philosophical differences contraindicate the use of linear models of change evaluation to accurately assess and understand the nature and process of change in the creative arts therapies (Aigen, 1991; Hayes et al., 2007; Chilton et al., 2015; Archibald and Gerber, 2018).

Our contention is reflected in Collins and Sayer’s (as cited in Hayes et al., 2007, p. 716) assertion that change in psychotherapy is a dynamic system which cannot rely upon more traditional linear methods of research to account for “intra-individual variability which traditionally has been viewed as ‘noise’ or error.” This paradigm shift allows for the inclusion of “dynamic and dialectic interactive process between these multiple intra/inter psychic and intersubjective realities” (Gerber, 2016, p. 656) representing the idiosyncratic vigor of pluralistic human phenomena and reliant upon “… the coexistence and dialectical tensions between levels of consciousness, temporality, and spatiality” (Archibald and Gerber, 2018, p. 3). This view allows for the creation of more interdependent, multi-dimensional mechanisms that are textural and descriptive rather than reductive and measurable. Such a paradigm “contributes to the development of a creative philosophical frame foundational for both an art[s] therapy theory as well as a research mentality and methodology the purpose of which is the generation of new knowledge (Johnson and Gray, 2010; Johnson, 2015; Gerber, 2016, p. 656).”

### Dynamic Systems of Change

Based on our research findings and this suggested paradigm shift we might conceptualize mechanisms of change, within the creative arts therapies, as dynamic systems of relational, imaginative, and dialectical phenomena the interaction of which transforms perception, emotion, relationships, and behaviors. In conceptualizing and visualizing how our primary thematic phenomena jointly form systems of change, we arrived at a few preliminary proposals. We propose three dynamic systems of transformation in which the primary and modifying thematic phenomena are aligned in different configurations and dynamic relationships (Figures 1–3). The three systems might be named the kinetic mobile system, the figure/ground system, and the orbital system. Although similar these models do vary with regard to the juxtaposition and inter- relationship of each phenomena, the degree and type of movement and dynamic interaction between and amongst the phenomena, and consideration of the requisite balance between essential chaos and organization related to implications for dynamic change within the creative arts therapies.

#### Kinetic Mobile System

In the kinetic mobile system of dynamic transformation, the themes are conceptualized as shapes that are connected by bi-directional arrows or invisible hanging wires. Each shape is carefully positioned relative to its the other familial themes and each is considered to be of relative equal weight and size. In this system, all of the parts are in constant motion in relation to one another creating infinite combinations, within and beyond their familial themes, of interactive dynamic encounters, collisions, confrontations, ruptures and ultimately resolutions. The dialectical rupture and resolution shape and the arts-based expression shape are positioned at the top and bottom of the mobile since, although not conceived as linearly related, are often considered to be pivotal as both initiators and holders of change. Relational attunement is positioned as central to moderating between the arts based expressive process and the dialectical rupture and resolution. The kinetic mobile model is multi-dimensional allowing for both this strategic positioning but also the possibilities of infinite other unpredictable juxtapositions in a cycle of change so that each relational and imaginational rupture sets off a new relational, imaginational creative resolutions. In this system the dynamics are emergent, unpredictable and cyclical –at any point in this system the chain reaction will be initiated and move through various phases. This system perhaps most accurately reflects delicate balance between chaos and organization and the potential for destruction or creativity, that is central to the resolution of the inherent dialectical relational arts-based processes contributing to a systems of change in the creative arts therapies.

#### Figure/Ground System

The figure/ground system of dynamic transformation from our study re-configures the primary and modifying familial themes in terms of contextual or conditional phenomena as the necessary background or holding environment for the more dynamic interactive or moving parts in the foreground. In this model, the two primary themes of arts-based expression and relationship and intersubjectivity are viewed as more contextual conditions essential for the emergence of dialectical rupture and resolution which is, in the intermediary ground, conceived as pivotal to change relative to interaction with the other phenomena. The other phenomena, although grouped in their thematic families, are also conceptualized as active and interactive in and around each other and the contextual conditions. In this model, instead of all of the parts randomly moving there are some phenomena that are conceptualized as stabilizers or holders so that the other parts can freely move around. These factors are the arts-based, relationship and intersubjectivity phenomena which are generally considered to be the essential and constant environmental factors central to change in the creative arts therapies. However, it should be noted that these contextual phenomena include multiple kinetic phenomena that might, under differing conditions, influence the degree of stability or rupture thus effecting the dynamics of the whole system. Relative to our musings about the degree and interaction between chaos and organization, this model attempts to provide a more intentional equanimity and delicate balance between the variability and stability of the phenomena with the understanding that this balance can be disrupted at any moment and under any conditions.

#### Orbital System

In the orbital system all major thematic phenomena are compressed into larger inclusive categories and visualized as equal in size and proximity from each other orbiting around and mediated by a bi-directional center. In this system, as in the others, there are multiple pathways for these phenomena to interact and influence each other for the purpose of informing change but perhaps in this system the possible combinations are more limited. This system appears simpler and less chaotic with less moving parts and limited pathways of interaction. The question arises as to how the simplification, organization, and restriction of possibilities influences the dynamism of these systems of change. In this case we have to critically evaluate if order and simplification sacrifices the essential ontological and epistemic nature of the phenomena and the value of human experience necessary for change in the creative arts therapies. This is an important consideration as we move toward exploring the most authentic systems of change in creative arts therapies. This lead us into considering methods and approaches to evaluating these findings and emergent systems of change.

Of course, these are very preliminary ideas and conceptualizations ripe for further creative discourse and investigation. The additional creative development might benefit from both construction of three dimensional actual and arts-based models to further study the systems of interaction combined with elicitation and documentation of the experiences of actual humans to contribute to the more totalistic understand these transformative phenomena.

### Evaluation

In proposing these dynamic systems of change, which are based both in our data and in psychotherapy and creative arts therapies theory and research, the questions arise as to how we would evaluate these dynamic systems as mechanisms of change; and, if they will contribute to our understanding of what change is and how it occurs in the creative arts therapies. Even though outside of the scope of this phase of the study, these questions warrant a momentary consideration relative to the implications for rigor, credibility, and epistemic authenticity in both research and clinical practice. Implicit in the paradigm shift from a post-positivist to a dialectical aesthetic intersubjective perspective (Chilton et al., 2015; Johnson, 2015; Gerber, 2016) is the construction of methods to evaluate the nature, qualities, and dynamics of these phenomena individually and interactively using epistemically comparable modes of assessment.

In qualitative research and arts-based research there are approaches to evaluating credibility and authenticity of similarly regarded phenomena (Barone and Eisner, 2012; Leavy, 2015). For instance, Barone and Eisner (2012) offer evaluative concepts for arts-based research such as incisiveness, concision, and evocation and illumination all of which relate to the aesthetic, emotional, intuitive, communicative, and relational qualities of arts-based expression while being mindful of rigor and authenticity. Perhaps there are parallel evaluative approaches using such concepts as applied to the evaluation of these phenomenological experiences in both research and clinical practices. Our evaluation processes would most likely consist of critical reflection and discourse, rich textural and textual description, and arts-based responses created and shared amongst participant/researchers and perhaps with an audience. Although these are just very preliminary and nascent ideas which require more thought and development it is necessary to begin thinking about them as we construct this dynamic system of transformative elements and consider ways in which we might understand their implications for research and clinical practice.

### Limitations and Implications for Future Research

The results of this study are preliminary and naturally include multiple limitations that both relate to the findings but also illuminate new directions for future research. The limitations and implications for future research cohabit the same dialectical spaces and relate to the nature of the analog study, the diversity, distribution and number of records reviewed, the importance and impact of the multiple perspectives of the coders, the nature and clarity of the coding system, and the noticeable absence of references to the transferential relationships.

The first limitation relates to the analog data which was collected from a laboratory setting simulating conditions parallel to the creative arts therapies experience. Although this is a limitation, since it is not data from actual creative arts therapies treatment, it could also conceivably be a benefit. In this analogous laboratory setting, the participant/researchers still experienced and expressed a range of perceptual, emotional, imaginal, relational, and behavioral phenomena similar to those of an actual therapy session. Additionally, since the key informants were both creative arts therapists and doctoral student participant/researchers, they were accustomed to the psychological repercussions inherent in creative arts therapies encounter. Therefore, they were able to experience, tolerate, and observe the anxieties, uncertainties, frustrations, joys, insights, and resolutions of this parallel situation. In addition, they were also able to document, analyze, articulately describe, and represent their experiences using arts-based and textual methods. The limitation resides in the transferability of these findings to actual creative arts therapies sessions representing multiple disciplines, settings, and populations. Perhaps upon further investigation and refinement of the results these systems of transformation might be studied in varying treatment contexts.

The next limitation relates to the diversity, distribution, and number of records relative to understanding the nature and progression of transformative phenomena over time as well as across and within disciplines. We initially selected 16 study records but ultimately only used eight for this preliminary study. The use of fewer records was based on our decision to conduct this preliminary pilot phase of the study in which we could test and evaluate the coding system, the inter-coder alignment, and adjust both as necessary for the next study phase. The limitation of using the fewer records is that we did not get the distribution we would have liked across the different yearly cohorts, the course progression, and creative arts therapies disciplines. A more even distribution data across cohort and discipline would provide breadth and diversity in all patterns of evidence as well as an analysis of discipline specific patterns of data and progressive responses over time. We did retrieve some promising preliminary data relative to the increasing frequency and appearance of particular phenomena in the progressive courses over time which may be relevant to our study of transformation, however, these data require further exploration. One interesting finding from these progressive frequencies was, that in contrast to the increase in all thematic phenomena, the sensory/kinesthetic/embodied knowledge theme decreased ever so slightly. One interpretation of this change could be that the increase of relationship, intersubjectivity, consciousness and open communication in the group diminished the need for and prevalence of more unconscious modes of knowing and communication—imagination or fantasy about others is transformed into knowledge, creative and open expression. Of course, there are multiple other confounding factors that could explain this finding and require examination before we can affirm this postulation; but, it provides interesting musings for future research.

In reviewing, analyzing and reflecting upon the data for this study, we became acutely aware of an additional limitation as well as a potential source of rich data related to the diverse perspectives of students and instructors. In particular, we were interested in the hierarchical relational phenomena that are parallel and central to transformation within the therapeutic relationship in the creative arts therapies process. Of note is the fact that these data were retrieved from a course in which students have concerns about being evaluated by the instructors, therefore, openly addressing their experience of this relationship in their culminating assignments posed a significant risk. Within this context, it is not surprising that there was minimal explicit reference to the real or imagined relationship between the student participant/researchers and the faculty participant/researchers in the records we reviewed. The stunning absence of reference to this hierarchical relationship is relative to the challenges of articulating these transferential phenomena with their associated real, perceived, and imagined scenarios and implications in both the classroom and psychotherapy setting. Further exploration of the specific ways in which these hierarchical relational mechanisms interact with our dynamic systems of change is warranted.

There were two major limitations relative to the coding system. First the definitions were in some cases awkwardly worded and consequently challenging to interpret which may have influenced the inter-coder alignment. Although we had a relatively high occurrence of inter-coder alignment, fine tuning these definitions might strengthen that alignment and contribute to concision and accuracy. Second, there was significant overlap between some of the coding categories. Therefore, even though some codes were not used frequently or at all, elements of those codes were implicitly represented in other codes (e.g., the parent category of reflection not coded but reflection was central to the resolution of dialectical ruptures). To address this issue, using the results of the first phase of the study we can rework the definitions for purposes of clarity, elimination, or amplification of overlap. We also might want to conduct another review and coding using more of an inductive process to identify additional thematic trends emergent from the text for comparison to and integration with the deductive categories for a more comprehensive and authentic reflection of the data.

Finally, in continuing our investigation into these dynamic interactive systems of transformation we hope to develop methods to evaluate if and how people change relative to these powerful human relational, imaginative, and dialectical experiences within the creative arts therapies. Now that we have defined what we believe are formative transformative phenomena and dynamic interactive systems of change we can begin to involve more stakeholders in interviews, focus groups, and or analog laboratory experiences to explore the credibility and authenticity of these preliminary results.

## Conclusion

The purpose of this study was to examine the dynamic and interactive factors that might be considered mechanisms of change in the creative arts therapies. We identified three primary thematic transformative phenomena of change with their interactive modifiers that acting in concert with each other form dynamic systems of change in the arts therapies. We suggest that dynamic systems of change are more relevant to the underlying epistemological and ontological foundations of the arts therapies than linear, causal and measureable mechanistic approaches. As we proceed into our next phase of the study we need to re-evaluate our coding categories and procedures, continue to develop inter-coder alignment protocols, critically evaluate the influence of the differing student/faculty perspectives, and expand on methods of evaluating the authenticity and credibility of these dynamic transformative systems.

## Author Contributions

NG conceived, proposed, researched, and wrote the article. KB and NP did the coding for the study. KB and CB worked on the literature review.

## Conflict of Interest Statement

The authors declare that the research was conducted in the absence of any commercial or financial relationships that could be construed as a potential conflict of interest.
